# Clinical Presentation of Frontal Sinus Squamous Cell Carcinoma in the Dog and Response to Treatment With Radiation Therapy in Eight Dogs

**DOI:** 10.1111/vru.70000

**Published:** 2025-01-05

**Authors:** Pietro Loddo, Luca Schiavo, Jane Dobson, Ola Marcinowska

**Affiliations:** ^1^ Queen's Veterinary School Hospital Cambridge University Cambridge UK; ^2^ Department of Oncology Service Davies Veterinary Specialist Higham UK; ^3^ OnkolVet Veterinary Clinic Opole and Poznan University of Life Science Poznań Poland

**Keywords:** canine, coarse fractionation, dog, frontal sinus, radiation therapy, squamous cell carcinoma

## Abstract

Primary frontal sinus squamous cell carcinoma (PFSSCC) represents a rare disease in dogs, and there is a general paucity of information in the current veterinary literature regarding its presentation and response to radiation therapy.

The objective of this retrospective observational study was to describe a series of dogs diagnosed with PFSSCC and report their response to radiation therapy.

Medical records of dogs with a diagnosis of PFSSCC were reviewed. Data collected included signalment, presenting complaint, clinicopathologic and diagnostic imaging findings, treatment, therapeutic response, and date of death or last follow‐up.

Eight cases of PFSSCC in dogs were treated with radiation therapy at the authors' institution. Three of these dogs were treated with coarse‐fractionated radiation therapy. One dog was euthanized due to an unrelated cause 36 months after completing the radiation therapy. The second and third dogs survived 18 and 3 months, respectively, from the end of treatment to death due to PFSCC. Five further dogs were treated with a more fractionated protocol (Monday–Wednesday–Friday schedule). The median survival time for all patients was 7.5 months (range 2–36 months).

Despite the small number of cases and variation in the radiation protocols used, the treatment outcomes in these eight dogs suggest that radiation therapy is potentially a viable treatment option for dogs with PFSSCC and that coarse fractionation might be an appropriate approach if more finely fractionated protocols are not possible.

## 1 Introduction

Primary frontal sinus squamous cell carcinoma (PFSSCC) is a very rare neoplasm both in people and in dogs, with an incidence rate in humans of 0.009–0.03% [1, 2]. In people, PFSSCC was first described by Prawssud [3]. Human patients are traditionally treated by neurosurgeons and otorhinolaryngologists [4]. The treatment of choice is radical surgery with or without adjunctive radiation therapy, but the prognosis is usually poor due to most tumors being advanced at the time of presentation [5, 6].

In dogs, frontal sinus squamous cell carcinoma (SCC) is most often described as a local extension of nasal SCC [7, 8]. The treatment of choice for dogs with nasal SCC is radiation therapy with or without surgery [9, 10]. There are few reports of PFSSCC in dogs in the veterinary literature; three cases reported in 2012 were treated medically [11], and two more recent cases were treated surgically, one of which also received adjuvant chemotherapy [1, 12]. While the current veterinary literature data regarding sinonasal SCC suggest that this tumor tends to respond to radiation therapy (RT), there are no reports in veterinary literature that focused solely on outcomes in dogs with PFSSCC treated primarily with radiation therapy. The purpose of this article is to describe the clinical presentation and outcome in eight dogs with PFSSCC treated with radiation therapy as the main treatment modality. To the authors’ knowledge, this is the first report describing radiation therapy as the main treatment modality in the management of dogs with PFSSCC.

## 2 Materials and Methods

The medical record database of the authors’ institution was searched between 2004 and 2020 for dogs that presented with a facial deformity associated with the frontal region and clinical signs of being “head shy”. Dogs were included in the study if they were diagnosed with PFSSCC by means of cytology and histopathology and if imaging results were consistent with a primary lesion of the frontal sinus. All patients included in the study received RT as their primary treatment. Dogs that, subsequently or during treatment with RT, received treatment with chemotherapy or tyrosine kinase inhibitors were not excluded from the study. Patients who were treated with concomitant symptomatic treatment (e.g., nonsteroidal anti‐inflammatory drugs [NSAIDs]) were not excluded from the study. Patients were excluded from the study if cytological or histopathological results were indicative of neoplasia other than squamous cell carcinoma and if there was an invasion of the nasal cavity and the tumor's origin could not be unequivocally defined to be the frontal sinus.

The staging was performed by applying the modified Adam's system [13]. Due to the anatomical position of PFSSCC, involvement of the cribriform plate is less likely to be apparent, and in the use of lysis of the calvarium, this was also considered stage 4. Patients were considered free of loco‐regional metastatic disease or distant metastatic disease if submandibular and retropharyngeal lymph nodes had normal imaging appearance and if no pulmonary lesions could be identified.

Tumor response to treatment was defined as complete response (CR), partial response (PR), stable disease (SD), and progressive disease (PD) as defined by RECIST guidelines. In the cases where follow‐up imaging results were not available, this was based on manual measurement of the visible lesion alone.

Survival time was calculated from the last RT fraction to the death of the animal from any causes.

The first follow‐up clinical examination to assess for potential acute side effects of radiation therapy and initial response to treatment was performed 2 weeks after completion of radiation therapy at the referring veterinary practice. Six to eight weeks after completion of the radiation therapy protocol, each patient was assessed at the authors’ institution. For longer‐term follow‐up, the owners were contacted by telephone or e‐mail for an update on their dog's status. Alternatively, dogs were re‐examined at the authors’ institution, or in some cases, the referring veterinary practices were also telephoned in order to obtain information about the patient's clinical progress.

Survival time was calculated from the end of radiation therapy to the date of death, regardless of cause.

## 3 Results

### 3.1 Patient Characteristics

Detailed presenting signs and signalment details are provided in Table 1. Eight dogs were included in the study, referred to as Dog 1 to 8, in chronological order.

**TABLE 1:** Case summary.

Case	Breed and signalment	Presenting signs	Diagnostic imaging and stage according to modified Adams stage	Diagnosis
Dog 1	6‐year‐old, M, Irish Water Spaniel	Extensive 3 × 3 cm mass, bulging from the right frontal sinus area with right exophthalmos	MRI, extensive soft tissue intensity in the right frontal sinus with invasion into the right orbit with lateral displacement of the eye; invasion and destruction of an area of the cranium; caudal extension to the level of the tympanic bulla Stage 4^a^	Incisional biopsy and histopathology: squamous cell carcinoma (SCC)
Dog 2	12‐year‐old, FN, Jack Russell Terrier (JRT)	Bony mass over the left frontal sinus	CT, extensive, mineralized, soft tissue attenuating mass of the left frontal sinus associated with marked sclerosis and left frontal sinus bone lysis; marked lysis of the frontal sinus bone adjacent to the orbit Stage 4	Incisional biopsy and histopathology: SCC
Dog 3	6‐year‐old, MN, Mastiff cross	Extensive mass in the region of the right frontal sinus measuring 4.8 × 6.1 cm	CT, large soft tissue attenuating mass of the right frontal bone, with marked cortical lysis; involvement of dorsal wall of the right orbit, dorsomedial aspect of the right retrobulbar space, causing a slight ventral displacement of the right globe; invasion of the dorsal aspect of the right frontal sinus Stage 4	Incisional biopsy and histopathology: SCC
Dog 4	6‐year‐old, FN, Labrador Retriever	Rapidly growing mass in the right frontal sinus region, measuring approximately 6 × 5 × 2.5 cm; pain on opening the jaw	CT, large soft tissue attenuating mass in the right frontal sinus with marked frontal and parietal bone lysis, with invasion into cranial vault causing compression and deviation of the dura and cerebral cortex Stage 4	Incisional biopsy and histopathology: SCC
Dog 5	5‐year‐old, MN, Rottweiler	Mass in the right frontal sinus, measuring approximately 5 × 5 × 2 cm	CT, soft tissue attenuating mass of the right frontal sinus with invasion of the adjacent frontal bone Stage 4	Incisional biopsy and histopathology: SCC
Dog 6	7‐year‐old, MN, Border Collie	Extensive mass in the left frontal sinus region, measuring approximately 6 × 4.5 cm	CT, large soft tissue attenuating mass of the left frontal sinus; with marked frontal bone lysis and extension of the mass into the dorsal left retro‐bulbar space and dorsoventral aspect of the left nasal cavity Stage 4	Incisional biopsy and histopathology: SCC
Dog 7	6‐year‐old, FN, Jack Russel Terrier	4 × 2.5 cm, fast‐growing mass, over the frontal sinus region. Pain at palpation of the mass	CT, mostly left‐sided soft tissue attenuating mass lesion. Associated with lysis of the frontal bone, frontal sinus septum dorsal cribriform, and calvarium Stage 4	Cytology: SCC
Dog 8	9‐year‐old, ME, Border Terrier	Left‐sided frontal sinus, fast‐growing mass. Measuring 4.2 × 4.5 cm Bilateral epiphora	CT, left frontal sinus soft tissue attenuating mass lesion. Calvarium lysis was present with mild intracranial extension Stage 4	Cytology: SCC

^a^ Stage 4 by Adams et al [13]. defines Stage 4 nasal tumors as having lysis of the cribriform plate. As dogs with frontal sinus SCC are unlikely to show cribriform plate involvement, and all eight dogs included in the current study had calvarium lysis, it was decided to qualify all eight dogs as Stage 4.

The median age of the patients was 6 years (ranging from 5 to 12 years).

Breeds included two Jack Russel terriers, an Irish Water Spaniel, a Mastiff crossbreed, a Labrador retriever, a Rottweiler, a Border collie, and a Border terrier.

**FIGURE 1:**
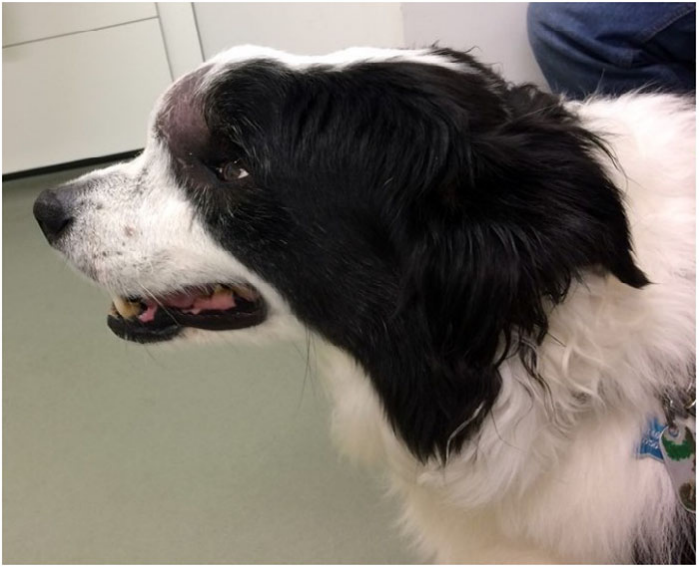
A–B, Dog 3: A, Transverse CT image of large soft tissue attenuating mass of the right frontal sinus with marked cortical bone lysis. B, dose distribution to the isocenter shown on the transverse CT view of the dog's patient.

All dogs presented with facial deformation and a hard, rapidly growing mass over the frontal sinus region (Figure 1). Right‐sided exophthalmos, bilateral epiphora, and pain in opening the jaw were noticed at presentation, respectively, in Dog 1, Dog 8, and Dog 4. In one patient (Dog 7), pain could be elicited upon palpation of the mass. In the remaining four cases, a degree of discomfort related to the presence of their mass was reported; however, this was not objectively quantified by pain scoring in any of the cases.

### 3.2 Tumor Characteristics and Staging Results

Detailed tumor characteristics and staging results are provided in Table 1.

**FIGURE 2:**
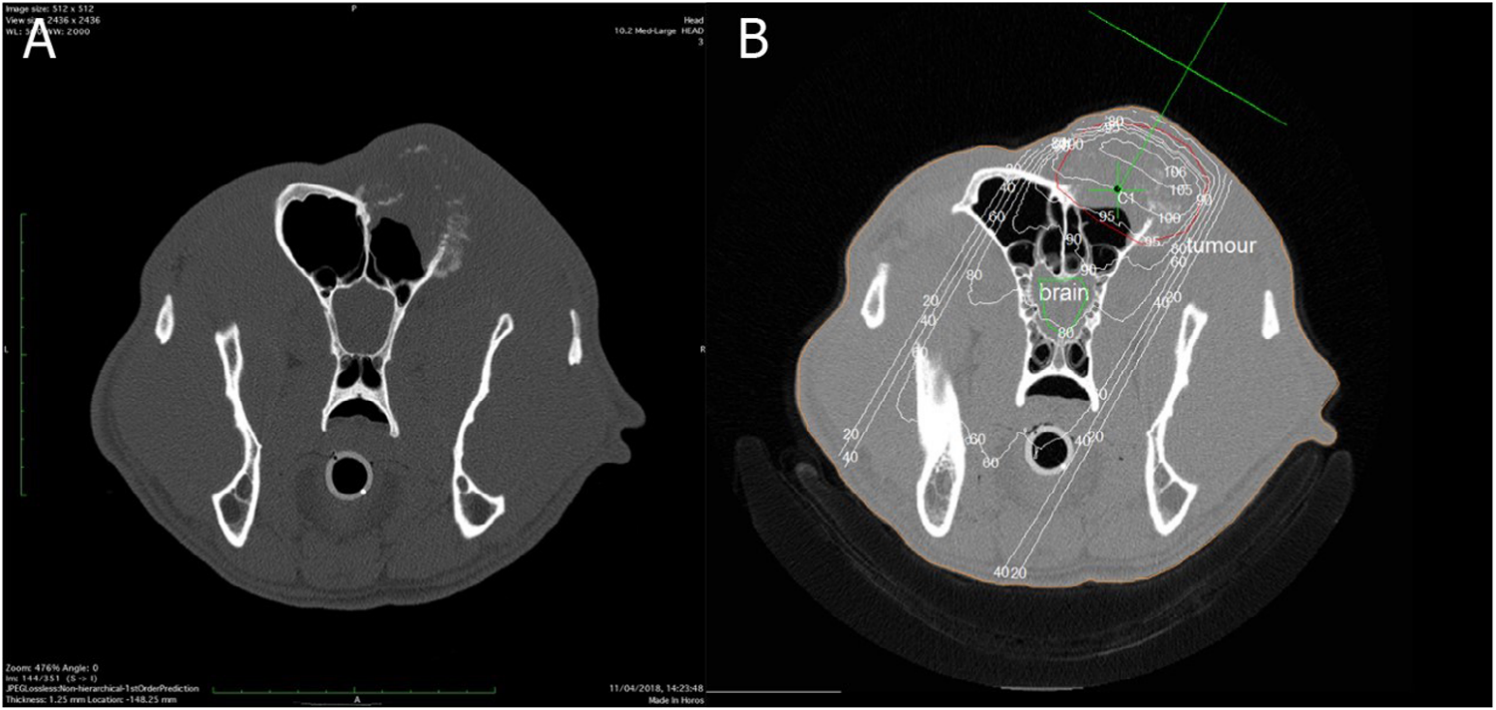
A, B, Dog 4: A, Dose–volume histogram (DVH) demonstrating the percentage dose delivered to the fractional volume of the: GTV (gross tumor volume), the brain, and the eyes. B, Sagittal view of the radiotherapy treatment plan.

All dogs had advanced imaging performed as part of their staging examination (Figure 2). All but one (Dog 1) had a CT of the head and neck; Dog 1 had an MRI of the head and neck. All dogs were classified as clinical stage 4, having either lysis of the cribriform plate or the calvarium. In all cases, the local regional lymph nodes were normal in size.

Thoracic imaging was available in all dogs but 2 (Dog 2 and 7). In all cases, this was provided by a CT scan, and there was no evidence of pulmonary metastatic disease.

External mass measurements were performed manually in the majority of the dogs, and the measurements are provided in Table 1. Two dogs (Dog 5 and 4) had further advanced imaging (one CT and one MRI, respectively) as part of their follow‐up assessment.

Ophthalmologic examination was not routinely performed prior to radiotherapy treatment. Treatment with a variety of NSAIDs was commenced in all dogs prior to and continued throughout the radiation therapy.

### 3.3 Radiation Planning and Treatment Parameters

All dogs were treated with external beam megavoltage (MV) radiation therapy delivered either with a 4 MV (Dynaray, Ray Technologies) or 6 MV (Varian, Clinac DMX‐ Varian Medical Systems UK Ltd.) linear accelerator. Neither linear accelerator was equipped with a multi‐leaf collimator. Hence, all of the treatment fields were rectangular or square, as defined by the collimator jaws of the treatment machine.

Treatment plans were calculated manually or using a computer‐based planning system (Addenbrookes Radiation Planning System [ARPS]). Six dogs were treated with 6 MV photons, and two dogs were treated with four MV photons. Scheduling, dose per fraction, and total dose delivered are detailed in Table 2.

**TABLE 2:** Radiotherapy details and outcome.

Case	Radiotherapy unit and protocol	Response	Toxicity	Outcome	Chemotherapy
Dog 1	Dynaray 4 MX photon 4 fractions of 8.5 Gy One fraction weekly. Total dose 34 Gy Manual plan	Complete response (CR) at the end of radiotherapy (RT)	Acute:VRTOG Grade 1 alopecia around the right eye and right cheekVRTOG Grade 2/3 right‐sided conjunctivitis Right eye lens cloudy at 6 weeks	Progressive disease (PD) at 3 months post‐RT, resulting in euthanasia	None
Dog 2	Dynaray 4 MX photon 4 fractions of 8 Gy One fraction weekly. Total dose 32 Gy Manual plan	Partial response (PR) at end of RT.	Acute: VRTOG Grade 1, left eye conjunctivitis at end of RT (week 4); mild inflammation around sclera at week 6 Marked mandibular and temporal muscle atrophy noticed 6 weeks after last fraction was delivered Late: left eye cataract at 6 months VRTOG Grade 2 left‐sided keratoconjunctivitis sicca (KCC) at 6 months. Sunken left eye at 6 months	PD and euthanasia 18 months post‐RT	None
Dog 3	Varian 6 MV photons 4 fractions of 8.5 Gy. One fraction weekly Total dose 34 Gy. 3D computer plan^a^	PR at end of RT (week 4) and CR at 6 week assessment.	Acute: None Late: None	Euthanasia was performed 36 months post‐RT due to noncancer‐related causes	None
Dog 4	Varian 6 MV photon 10 fractions of 4 Gy Treatment on a Monday–Wednesday–Friday schedule Total dose 40 Gy 3D computer plan	Stable disease (SD) achieved during RT	Acute: VRTOG Grade 1 right‐sided conjunctivitis VRTOG Grade 2 moist dermatitis of skin around right eye week 4 and 6.	PD at 3 months post‐RT‐MRI head performed, extensive mass eroding most of the frontal bone	Carboplatin at first RT, discontinued due to VCOG Grade 1/2 neutropenia; Toceranib phosphate (Palladia) at 2.75 mg/kg per os every other day
Dog 5	Varian 6 MV photon 10 fractions of 4 Gy Treatment on a Monday–Wednesday–scheduling Total dose 40 Gy 3D computer plan	PR at the end of RT which remained so for 7 months.	Acute: VRTOG Grade 1 mucositis and conjunctivitis at week 2, which progressed to Grade 3 mucositis and conjunctivitis at the end of RT VRTOG Grade 1 desquamation above right eye Mild right eye blepharospasm Mild clear discharge from right eye Late: None	PD 7 months after completion of RT. Toceranib phosphate was started and euthanasia was performed 6 weeks later	Carboplatin × 4. Toceranib phosphate (Palladia) at 2.75 mg/kg on Monday–Wednesday–Friday schedule
Dog 6	Varian 6 MV photon 10 fractions of 4 Gy Treatment on a Monday–Wednesday–Friday scheduling Total dose 40 Gy 3D computer plan	SD, no change of size of mass.	Acute: None Late: None	PD at 6 weeks post‐RT, euthanasia 2 weeks later	None
Dog 7	Varian 6 MV photon 6 fractions of 6 Gy Treatment on Monday‐Friday scheduling Total dose 36 Gy Manual plan	PR at the end of RT. CR achieved 4 weeks following RT	Acute: None Late: None	PTS at RV due to PD 7 months following the end of RT	Carboplatin 300 mg/m^2^ × 6. Toceranib, unknown dose, and scheduling
Dog 8	Varian 6 MV photon 6 fractions of 6 Gy Treatment on Monday‐Friday scheduling Total dose 36 Gy Manual plan	PR at the end of RT. CR achieved 4 weeks following RT	Acute: None Late: Seizuring 17 months post‐RT, causes were not investigated	PTS at RV due to seizure 17 months following the end of RT	Carboplatin 300 mg/m^2^ × 6

**FIGURE 3:**
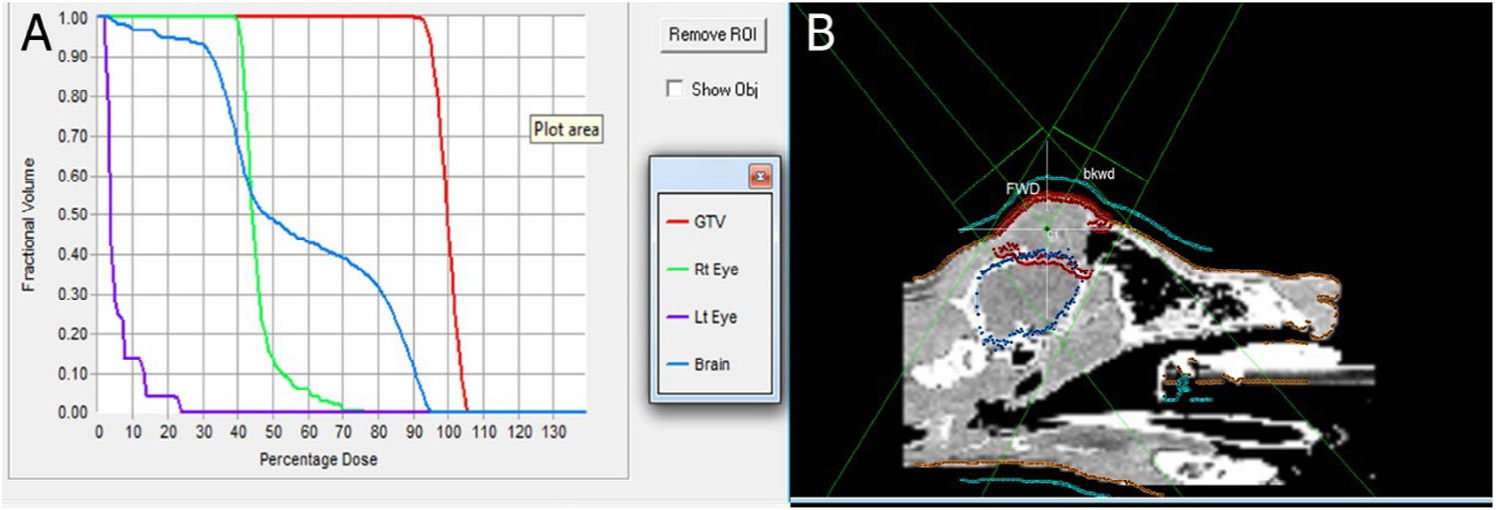
Photograph of dog 6; typical appearance of primary frontal sinus squamous cell carcinoma. A hard, firm mass is causing severe facial deformation, in this picture swelling at the level of the frontal sinus can be appreciated.

For the computer‐planned treatments, the outlines of the head, tumor, and organs at risk (OAR), namely the eyes and brain, were contoured on the CT data. The isocenter was positioned visually at the center of the tumor mass. The gross tumor volume (GTV) was defined by contrast‐enhancing areas within the CT data. The planning target volume (PTV) was defined by an expansion margin of 5 mm in all dimensions around the GTV to account for positioning errors as well as any variations of organ, tumor, and patient movement, inaccuracies in beam and patient setup, and any other uncertainties. Plans were evaluated by the same radiation oncologist (J.D.) using isodose lines and dose–volume histograms for the tumor volumes and OARs (Figure 3).

For treatment delivery, patients were anesthetized and positioned in sternal recumbency. The anesthetic protocols consisted of premedication with a short‐acting agent that varied occasionally depending on the animal's health and temperament but, in most cases, was atropine sulfate (Atrocare, Animal care Ltd.) and alfentanil hydrochloride (Rapifen, Janssen Cilag Ltd.). Propofol (VetoFlo Norbrook Laboratories Ltd) was used to induce anesthesia, which was then maintained with isoflurane (Isoflurane, Baxter Healthcare Ltd) or sevoflurane (Sevoflo, Abbott Laboratories Ltd.) and oxygen intermittent positive pressure ventilation. These were the standard anesthetic approach for radiation therapy treatment of patients at the authors' institution during patient accrual. Anatomical landmarks of the head, as well as surface‐to‐skin distances, as calculated by ARPS, were used to verify the dose was to be delivered to the tumor volumes as intended. Portal imaging for verification of patient setup was not performed in any of the cases. Bite blocks and vacuum bag positioning cushions were used in three dogs. Nonsteroidal anti‐inflammatory drugs were prescribed at clinicians’ discretion.

The reporting of radiation therapy treatments for the eight patients, when possible, followed the guidelines described by Keyerleber [14]; however, due to the retrospective nature of this study and manual planning in some cases, the necessary information to fully comply with the guidelines was not always available. Details on exposure of region of interest, GTV, PTV, and CTV are detailed in Table 3.

**TABLE 3:** 3D planning available data on region of interest, gross tumor volume (GTV), clinical target volume (CTV), and planning target volume (PTV).

Patient's ROI		GTV	Right eye	Left eye	Brain	CTV	PTV
Dog 3^a^	Max	106.9%	98.5%	47.7%	91.6%	Data not available	Data not available
	Min	8.2%	7.9%	3.9%	5.3%	//	//
	Mean	96.2%	70.2%	7.5%	72.5%	//	//
	Volume (CC)	97.6	5.2	5.6	97.6	//	//

		GTV	Right eye	Left eye	Brain	CTV	PTV
Dog 4	Max	105.4%	79.9%	34.7%	97.4%	Data not available	105.4%
	Min	89.9%	39.5%	2.3%	3.4%	//	72.9%
	Mean	99.7%	46.0%	5.7%	57.8%	//	98.5%
	Volume (CC)	44.7	5.4	5.2	116.6	//	78.6

		GTV	Right eye	Left eye	Brain	CTV	PTV
Dog 5	Max	106.1%	104.4%	50.3%	98.1%	106.9%	107.3%
	Min	93.7%	95.6%	38.2%	3.6%	75.8%	69.1%
	Mean	102.4%	100.7	43.5%	40.5%	101.5%	100.4%
	Volume (CC)	65.8	6.3	6.4	98.4	135.8	220.0

		GTV	Right eye	Left eye	Brain	CTV	PTV
Dog 6	Max	Data not available	79.3%	98.6%	106.3%	Data not available	116.9%
	Min	//	5.2%	7.3%	0.0%	//	49.6%
	Mean	//	15%	43.7%	11.0%	//	100.0%
	Volume (CC)	//	5.5	4.4	88.6	//	66.5

^a^ Dog 3 treatment planning was 3D, computer generated, and intended for 6 fractions. However, following the delivery of the first fraction the owner decided on a less intense protocol of 4 fractions. Data reported in Table 3 are adjusted for these changes.

Dog 1 was treated in sternal recumbency using a single 13 × 8 cm dorsoventral (DV) beam. Sandbags under the chin were used to elevate the head with the bridge of the nose in a horizontal position. No mouth bite system, dental mold, vacuum bag, eye blocks, or bolus were used for this patient.

Dog 2 was treated in sternal recumbency, using a single 8 × 5 cm DV beam and sandbags under the chin, as for Dog 1, with a lead block to protect the left eye. No mouth bite system, dental mold, vacuum bag, or bolus were used for this patient.

Dog 3 was treated with a computer‐generated plan initially, which was applied at his first of an intended total of six fractions, but thereafter, the owners decided to continue with a palliative intent protocol with three further weekly fractions, all in sternal recumbency with sandbags under the chin. The first fraction used a single DV treatment beam measuring 10 × 7 cm, narrowed to 9 × 6 cm for the remaining fractions 2–4, with a lead block protecting the right eye. Flab bolus was also used. No mouth bite system, dental mold, or vacuum bag were used for this patient.

Dog 4 was in sternal recumbency using a positioning box, which was not fixed to the treatment couch. Two treatment beams were used, a somewhat rostrally angled DV beam of 6.9 × 6.4 cm and a somewhat caudally angled DV beam of 6.5 × 6.2 cm, convergent on the isocenter, delivered with a treatment couch at 270°. A source‐to‐surface distance (SSD) of 97.5 cm for the rostral and 96.9 cm for the caudal treatment fields was used. A mouth bite system, dental mold, and vacuum bag were used for this patient.

Dog 5 was treated in sternal recumbency. Two treatment beams were used: a left oblique beam of 9.9 × 8.1 cm with 45° wedge and a right oblique beam of 9 × 9.1 cm with 25° wedge, convergent on the isocenter, both with SSD of 95.8 cm. Lead blocks were used to preclude brain exposure, with 1 cm of bolus material used. A mouth bite system fixed to the treatment couch, dental mold, and vacuum bag were used for this patient. The mean, minimum, and maximum doses for PTV were 100.4%, 69.1%, and 107.3, respectively.

Dog 6 was treated in sternal recumbency. Four treatment beams were used: a left lateral beam of 5.8 × 4.6 cm, a left oblique beam of 11.1 × 4 cm, a right oblique beam of 10.9 × 4 cm, and a rostral beam of 5.3 × 7 cm, with SSDs of 97, 97.3, 97.2, and 96.3 cm, respectively, and 0.5 cm of bolus material was used. A mouth bite system was fixed to the treatment couch, and a dental mold and vacuum bag were used. No lead blocks were used.

Dog 7 was treated in sternal recumbency, using a single 4 × 5 cm DV beam, with sandbags to elevate the chin with the bridge of the nose in a horizontal position and 1 cm bolus material. No mouth bite system, dental mold, vacuum bag, or lead blocks were used.

Dog 8 was treated in sternal recumbency, using a single 5 × 4.5 cm DV beam with sandbags to elevate the chin with the bridge of the nose in a horizontal position and 0.5 cm bolus material. No mouth bite system, dental mold, vacuum bag, or lead blocks were used.

### 3.4 Tumor Response, Toxicity, and Survival Outcome

In 6 of the 8 dogs, a substantial reduction in the tumor mass was documented at completion of the radiation course, with 4 dogs (Dog 1, 3, 7, and 8) achieving a gross complete response (CR), 2 dogs a partial response (PR; Dog 2 and 5) and 2 dogs were classified as static disease (SD; Dog 4 and 6). All dogs were subsequently assessed for clinical response by clinical examination; additionally, two dogs had advanced imaging performed: Dog 4 had head MRI, and Dog 5 had thoracic and head CT. Six dogs were eventually euthanized due to tumor‐related causes, with survival times ranging from 2 to 18 months.

Dog 8 was euthanized 17 months from the end of radiotherapy treatment following the development of seizures. Seizure etiology was not investigated, and it is unclear if this is related to either tumor progression or late side effects associated with radiation treatment. Dog 3 was euthanized at the referring veterinary practice 36 months following treatment for noncancer‐related causes.

**FIGURE 4:**
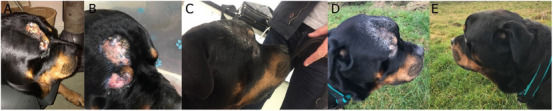
A–E, Dog 5: A, B, Moderate moist desquamation of the skin (VRTOG Grade 2) above the dog's right eye with Grade 3 RTOG mucositis and conjunctivitis of the right eye, at the tenth radiotherapy treatment; C–E, Pictures show the same patient with a progressive disease, 5 months after completion of the radiotherapy treatment.

Thorough ophthalmologic examinations were not routinely performed on the patients prior to treatment. However, all the patients had their eyes assessed as part of their clinical follow‐up examination, including Schirmer tear tests when indicated by suspicion of keratoconjunctivitis. Acute toxicities were documented in 4 of the 8 dogs during or at the end of treatment and included VRTOG (Veterinary Radiation Therapy Oncology Group) Grade I—II periocular alopecia and dermatitis (3 dogs) and Grade I—II conjunctivitis (4 dogs), the latter progressing to Grade III mucositis and conjunctivitis by the end of treatment in dog 5 (Figure 4). Most of these toxicities were mild and managed in the short term variably with systemic NSAIDs (e.g., Meloxicam) and topically with steroid‐based eye drops, (Maxitrol 0.1%, Novartis Pharmaceuticals UK Ltd.), Viscotears and fusidic acid/betamethasone topical cream (Isaderm, Dechra Veterinary Products). All resolved spontaneously within 3 – 6 weeks of completion of the radiation course.

Late toxicities were documented or suspected in 2 dogs; one dog developed a cataract with mild keratoconjunctivitis sicca (KCS) at 6 months postradiation. Another dog was euthanized due to a seizure, the cause of which was not determined. Two further dogs only survived for 3 months, which may be too soon following radiation for the development of late toxicity.

Four dogs (Dog 4, 5, 7, and 8) also received carboplatin (300 mg/m^2^) during and/ or after the radiation course, and two of these (Dog 4 and 5) also received toceranib phosphate at 2.75 mg/kg eod. One dog (Dog 4) only received one dose of carboplatin in conjunction with RT, and further treatments were not continued due to the development of a VCOG Grade 1/2 neutropenia. All other patients who were treated medically started treatment no sooner than 6 to 8 weeks from the end of RT.

Dog 5, 7, and 8 registered PR, CR, and CR, respectively, before adjunctive chemotherapy was started. Patient 4 had one dose of carboplatin 300 mg/m^2^ during RT, and SD was achieved.

Patients receiving adjunctive chemotherapy survived 3, 7, 8, and 17 months, while patients treated with sole RT survived 2, 3, 18, and 36 months.

The median survival time for the eight patients was 7.5 months; this ranged from 2 to 36 months.

## 4 Discussion

Squamous cell carcinoma confined strictly to the sinus is a very rare entity in both humans and companion animals. The purpose of reporting this small series of eight affected dogs is to highlight the potential role of radiotherapy in the management of this condition. Although the radiotherapy protocol and its delivery varied among the 8 dogs, 6 of the 8 dogs showed objective responses to the radiation, with 3 dogs surviving in CR for more than 17 months.

Radiation therapy treatment for primary frontal sinus SCC in dogs has not been reported per se, as in all veterinary publications, these tumors are classified as sinonasal tumors. It is well known that radiation therapy improves clinical signs, increases survival times, and is considered the treatment of choice for sinonasal neoplasms; however, the long‐term prognosis remains poor, with the median survival times of approximately 12 to 18 months following a full course, fractionated megavoltage radiation therapy [15, 16, 17].

Various radiation protocols for the management of sinonasal tumors are described in the veterinary literature. Coarse‐fractionated protocols with a large dose per fraction ranging from 4 to 10 Gy per fraction given weekly for 3–4 weeks to a total dose of 20–36 Gy result in good palliation of epistaxis and pain associated with nasal tumors, with 91.6–100% response rates for the duration of 3–14 months [18, 19]; Mellanby et al. [20, 21, 22].

Curative intent protocols delivering 2–3 Gy per fraction daily, totaling 48–54 Gy, and the use of intensity‐modulated radiation therapy (IMRT) have also been described and used in dogs with sinonasal tumors. Studies by Adams et al. [13], Bowles et al. [23], Hunley et al. [24], and Lawrence et al [25]. have reported median survival times ranging from 6.7 to 23 months.

Intensity‐modulated radiation therapy, characterized by its higher precision, allows for maximal dose delivery while minimizing exposure to healthy surrounding tissues. This precision is particularly beneficial for complex or irregularly shaped tumors. A retrospective multicentric study by Alvarez et al. [26] assessed IMRT‐based stereotactic radiation therapy in 17 dogs with nasal carcinomas, demonstrating clinical benefit in 88% of patients, with a median survival time of 563 days. Severe acute adverse effects were absent, but six patients experienced Grade 3 late side effects.

Additionally, Yoshikawa et al [27]. conducted a study on 182 dogs with nonlymphomatous sinonasal carcinoma, utilizing stereotactic radiation therapy. This approach yielded a disease‐specific survival time of 482 days. Seven percent of dogs developed oro‐cutaneous or naso‐cutaneous fistula, and 61% exhibited possible chronic rhinitis.

While not specifically targeting PFSSCC, these studies suggest that modern delivery methods could provide significant benefits in managing this condition. The increased accuracy of stereotactic radiotherapy holds promise for enhancing treatment outcomes for PFSSCC, a challenge to address anatomically with less precise radiation therapy approaches. Stereotactic techniques also facilitate the sparing of critical OAR, such as the eyes and brain, thereby improving treatment tolerability. Additionally, these methods may allow for the administration of higher total doses while reducing the number of treatment sessions, consequently lowering anesthesia‐related risks and minimizing patient clinic time. These factors underscore the potential of newer, more precise techniques as viable options for managing PFSSCC in dogs, warranting further investigation in future studies.

The cases in this report were treated with different radiotherapy protocols and total doses. Although 7 of the 8 dogs showed clinical benefit, the number of cases reported here is too small to draw any conclusions as to which approach might be most efficacious.

Radiotherapy protocols that include a higher number of fractions and the use of 3D planning are usually associated with improved outcomes; however, in this small series, of the three dogs that meet this criterion, one seemingly failed to respond (dog 6) and developed progressive disease only 6 weeks after the end of radiotherapy treatment, while a second patient (dog 4) achieved stable disease that lasted 3 months before progression of the disease was detected. This is an unexpected result that may have multiple explanations. These patients may have been affected by a more inherently aggressive form of the disease, and the clinician may have purposely selected a more intense protocol for the cases that had the worst presentation. Furthermore, in the absence of portal imaging verification, a risk of geographical miss can be as high as 36% of the cases, as described by Marks et al [28]. The use of portal imaging verification can enhance the accuracy of beam alignment and greatly decrease the risk of localization errors [29]. As such, localization and field design errors may have limited treatment efficacy in these cases, resulting in a worse outcome.

In terms of acute toxicity, the eye was the main organ at risk; 4 of the 8 dogs developed Grade 1–2 conjunctivitis, and 2 of these showed periocular alopecia/dermatitis, all of which resolved with symptomatic medical management. Only one dog, receiving 10 fractions, developed Grade 3 mucositis and conjunctivitis at the end of the treatment, and this was resolved within 1 month with symptomatic treatment. Four dogs showed no signs of acute toxicity. Five dogs did not survive long enough to fully assess late toxicity; the development of cataracts was suspected in one dog, which was euthanized due to progressive disease 3 months after completion of treatment. One dog, who survived 18 months, did develop KCS and a cataract in the ipsilateral eye by 6 months following completion of treatment. Keratoconjunctivitis sicca is a recognized complication of lachrymal gland irradiation in human radiation oncology, where a dose–response relationship between the maximum dose to the lacrimal gland and the development of KCS is established. A similar relationship has recently been reported in veterinary radiation oncology, where a minimum lachrymal gland dose for developing KCS was 23.75 Gy (Poirier et al.) [30].

The brain is a potential OAR, and one dog was euthanized at the referring veterinarian 17 months following the end of RT due to having developed a prolonged seizure. This was unfortunately not investigated, and so it was not possible to determine whether the seizure could be associated with tumor progression, late RT side effects, or unrelated to either.

In human patients, treatment options for paranasal sinus cancer are surgery, chemotherapy, and radiotherapy. Surgery for sinonasal malignancies in people has dramatically evolved over recent years which is due to the progressive application of intranasal endoscopic techniques, a decrease in indications for external approaches, continued advances in imaging techniques, and an increase in surgical expertise [31].

The extent of the disease in dogs and the close association of the tumor to the vital structures in the dog's head (i.e., eye, brain) make surgery technically challenging. Two veterinary reports describe surgical management and approaches; however, these were not without complications. One of them underwent tumor excision with partial craniectomy and chemotherapy (carboplatin/5‐fluorouracil) and continued to do well 16 months after surgery [12]. The second dog underwent superior orbitectomy as a sole treatment; he suffered from a local recurrence 12 months later, which was treated with carboplatin. The dog was euthanized 21 months following orbitectomy and 5 months after finishing the chemotherapy [32].

The utilization of conventional chemotherapy as the exclusive therapeutic approach for dogs afflicted by PFSSCC lacks well‐defined parameters within the veterinary literature, with limited data primarily available through case reports. The current body of evidence supporting its efficacy in PFSSCC is inadequate, highlighting the necessity for further comprehensive studies. In contrast, existing data on sinonasal SCC leans toward RT as the preferred treatment modality, speculation drawn from the available literature.

PFSSCC in dogs appears predominantly as a localized disease. Therefore, treatment modalities focusing on local control, such as RT, surgery, or a combination thereof, may prove to be more efficacious, aligning with findings in sinonasal carcinoma literature as a whole. In light of the limited role of chemotherapy as a standalone treatment, exploring its potential contribution within a multimodal therapeutic approach is a viable avenue for further investigation.

### 4.1 Limitations

There are a number of limitations to this study, the main one being the small number of cases. The radiation treatment protocols used in these eight canine patients were not standardized or uniform, which in part reflects the rarity of these tumors such that the eight cases reported here were treated over a period of 16 years, during which time the rapid development of veterinary radiotherapy has seen significant advances in treatment planning and delivery. It must be recognized that as a result of tumor volume, manual planning, and use of a single beam, which may result in poor dosimetric coverage of the PTV/GTV, some tumors may not have been treated to meet dose constraints currently applied to radiotherapy patients. However, this small series of cases does indicate a role for radiotherapy in the management of such tumors. Outcomes may be improved by better radiation treatment planning and more accurate treatment delivery. The radiotherapy machines used for the treatment of the eight dogs had no portal imaging; therefore, no patient verification was performed for any of the patients prior to any of the treatments; as previously discussed, this may affect clinical results and potentially increase the risk of geographical miss and side effects. There were no multileaf collimators built into the radiotherapy machine to aid in tissue sparing. Only one dog (Dog 5) had a re‐staging CT, and one dog (Dog 4) had a head MRI performed, with the remaining six dogs having had their tumor response assessed visually during a clinical examination. As such, tumor response, as defined by the RECIST guidelines, could not be performed precisely for all dogs. However, in all cases, the primary lesion was clinically apparent, and measurements were relatively easy to obtain. The follow‐up for the cases was by telephone conversation and/or e‐mail correspondence, limiting the ability of the authors to accurately evaluate the development of toxicity. This limitation was greater in the case of patients who survived longer and were more likely to develop late toxicity.

Four patients were also treated with adjunctive chemotherapy or TKI; this may have affected tumor response and survival time and acted as a confounding factor, precluding an accurate evaluation of clinical results that are inherent to RT alone.

Furthermore, in two cases, staging workup for metastatic disease was incomplete, and data regarding thoracic imaging could not be found or was not performed. Often, sinonasal carcinoma and sinonasal SCC are characterized by low metastatic potential. However, metastatic potential in the case of PFSSCC is yet not well defined, and this may have affected survival time in some patients.

## 5 Conclusion

This small case series suggests that radiation therapy has a role in the management of dogs with PFSSCC and that coarse fractionation might be an effective means of palliation if finely fractionated protocols are not possible. Outcomes may be further improved by better radiation treatment planning and delivery. The radiotherapy‐related side effects were mainly mild and manageable with medical/symptomatic treatment; cataract formation developed only in one dog. The addition of chemotherapy (carboplatin) and toceranib phosphate to radiation therapy should be evaluated in a larger cohort of dogs.

## Conflicts of Interest

The authors declare no conflict of interest.

## Ethics Statement

All relevant legal and ethical requirements have been met with regard to the humane treatment of animals in this case report. Informed consent for treatment and the use of data in clinical research was obtained. Patient care, in all cases, was in line with the current treatment standard of care.

## Previous Presentation or Publication Disclosure

Poster at ESVONC 2022.

## Reporting Checklist Disclosure

An EQUATOR network or other reporting checklist was not used.

## Data Availability

The data supporting the results of this study are available from the corresponding author upon reasonable request.
